# PPP1CC Suppresses Preadipocyte Differentiation in Chickens at Least Partly by Regulating NRF1 Expression

**DOI:** 10.3390/genes17040375

**Published:** 2026-03-26

**Authors:** Tingting Cui, Aicheng Zhang, Xifeng Zhang, Qingzhu Yang, Hongyan Chen, Xinyuan Li, Rongyan Huang, Lanlan Zhang, Weiwei Zhang

**Affiliations:** 1 College of Life Science and Agriculture Forestry, Qiqihar University, Qiqihar 161006, China; cuitingting.1985@163.com (T.C.); zhang_aicheng@163.com (A.Z.); dec_xf@163.com (X.Z.); yqzyg1123@163.com (Q.Y.); 03692@qqhru.edu.cn (H.C.); xinyuanlee@163.com (X.L.); hry_ok@163.com (R.H.); rainbowforever7@163.com (L.Z.); 2 Heilongjiang Provincial Key Laboratory of Resistance Gene Engineering and Protection of Biodiversity in Cold Areas, Qiqihar 161006, China; 3 Postdoctoral Research Workstation, Heilongjiang Academy of Agricultural Sciences, Harbin 150069, China

**Keywords:** adipogenesis, chicken preadipocytes, dephosphorylation, PPP1CC, protein–protein interaction

## Abstract

**Background:** Excessive abdominal fat deposition is a major challenge in the chicken farming industry, making it essential to elucidate the molecular mechanisms underlying chicken adipogenesis. Nuclear Respiratory Factor 1 (NRF1) has been reported to suppress chicken adipogenesis by downregulating peroxisome proliferator-activated receptor gamma (PPARγ) expression. Protein Phosphatase 1 Catalytic Subunit Gamma (PPP1CC) is a multifunctional phosphatase involved in various biological processes; however, its role in chicken adipogenesis remains unclear. **Objective:** This study aimed to investigate the functional role and underlying mechanism of PPP1CC in chicken preadipocyte differentiation. **Methods:** Co-immunoprecipitation (Co-IP) and immunofluorescence assays were performed to determine the interaction between PPP1CC and NRF1 in DF1 cells. Bioinformatic analysis predicted potential NRF1 dephosphorylation sites targeted by PPP1CC, based on which NRF1 mutants mimicking dephosphorylation were constructed. Phos-tag SDS-PAGE combined with Western blot analysis were used to verify PPP1CC-mediated dephosphorylation of wild-type NRF1. Dual-luciferase reporter assays were used to evaluate the effect of PPP1CC-mediated dephosphorylation on NRF1-regulated *PPARγ* P1 promoter transcriptional activity. ChIP-qPCR was employed to assess the occupancy of NRF1 to the *PPARγ* P1 promoter upon PPP1CC overexpression. The effect of PPP1CC overexpression was assessed on preadipocyte differentiation using Oil Red O staining and marker gene expression analysis. **Results:** PPP1CC interacted with NRF1 in both the cytoplasm and nucleus of DF1 cells. Overexpression of PPP1CC significantly promoted NRF1 dephosphorylation during oleic acid-induced preadipocyte differentiation and increased endogenous NRF1 expression. Moreover, dual-luciferase assays showed that while PPP1CC strengthened the inhibitory effect of wild-type NRF1 on *PPARγ* P1 promoter transcriptional activity, it exerted no additional suppression on the already low activity mediated by the dephosphorylation-mimicking NRF1 mutants. Consistently, ChIP-qPCR results demonstrated that PPP1CC overexpression enhanced the occupancy of NRF1 to the *PPARγ* P1 promoter. Functional assays revealed that PPP1CC overexpression significantly inhibited chicken preadipocyte differentiation. **Conclusions:** PPP1CC interacts with NRF1 and promotes its dephosphorylation, enhancing NRF1-mediated suppression of PPARγ transcription and ultimately inhibiting chicken preadipocyte differentiation. These results identify the PPP1CC–NRF1–PPARγ regulatory axis and provide a potential molecular target for controlling fat deposition in broiler chickens.

## 1. Introduction

Chicken has become the second-most-consumed meat in China after pork [1]. However, excessive abdominal fat deposition in broilers has emerged as a major issue that urgently requires resolution in breeding and production systems. A deeper understanding of the molecular mechanisms underlying adipose deposition in chickens would provide both a theoretical foundation and technical support for reducing excessive abdominal fat accumulation in broilers. Adipocyte differentiation, also known as adipogenesis, refers to the process by which fibroblast-like preadipocytes differentiate into mature adipocytes [2,3]. Despite extensive studies, the regulatory mechanisms governing adipocyte differentiation in broilers remain incompletely understood.

Adipogenesis is a complex biological process regulated by an intricate transcriptional regulatory network [4,5]. Numerous transcription factors involved in adipogenesis have been identified, including NRF1, PPARγ, C/EBPα, SREBPs, STAT5, FOXO1, KLFs, Krox20, PATZ1, GATA2, GATA3, and HES1 [4,5,6]. Among these factors, PPARγ and C/EBPα are considered the key regulators of adipogenic differentiation. These two transcription factors can mutually promote their binding to chromatin, synergistically regulating the expression of genes associated with adipogenic differentiation [7].

Post-translational modifications, particularly phosphorylation, can affect the function of transcription factors by altering protein stability, activity, subcellular localization, and interactions with other molecules [8,9,10]. Moreover, multiple cellular signaling pathways participate in the regulation of adipogenesis, including the TGFβ, BMP, insulin, IGF, Wnt, PPARγ, Hedgehog, Notch, FGF, DLK1/PREF1, AMPK, and MAPK signaling pathways [11]. Transcription factors, post-translational modifications, and signaling pathways collectively contribute to the regulation of adipogenesis. However, the mechanisms through which these regulatory components interact to control chicken adipogenesis remain poorly understood. Elucidating these interactions would improve the understanding of adipose tissue development in chickens and provide theoretical insights for controlling excessive fat deposition.

Our previous study demonstrated that the transcription factor NRF1 acts as a negative regulator of chicken adipogenesis. NRF1 can directly bind to the P1 promoter of the chicken *PPARγ* gene, downregulate *PPARγ1* mRNA expression, and inhibit chicken adipogenesis [12]. NRF1 is also associated with various biological processes, including diabetes, cancer cell proliferation and migration, apoptosis, signal transduction, mitochondrial biogenesis, cellular inflammation, and adipogenesis [13]. Previous studies have shown that NRF1 expression is closely related to its phosphorylation status, and phosphorylation can exert bidirectional regulatory effects on NRF1 expression [14,15]. For example, in mouse neuronal cells, the *ATM* gene induces phosphorylation of NRF1 at Thr259, promoting NRF1 protein dimerization, enhancing its nuclear translocation, and increasing *NRF1* mRNA and protein levels, stimulating mitochondrial biogenesis by upregulating nuclear-encoded mitochondrial genes [14]. In mouse germ cells, NRF1 has been identified as a substrate of protein kinase CDK2. CDK2 interacts with NRF1 and phosphorylates it at Ser318, leading to reduced NRF1 protein levels and decreased NRF1 binding to the promoter of its target gene, *Ehmt1*, thus regulating H3K9 methylation during meiotic prophase [15].

PPP1CC belongs to the protein phosphatase 1 (PP1) subfamily and participates in the regulation of multiple biological processes, including cell proliferation, apoptosis, spermatogenesis, DNA repair, cancer cell radioresistance, insulin resistance, and meat color variation [16,17,18,19]. Knockdown of PPP1CC in spermatozoa significantly reduces porcine sperm motility and increases cell apoptosis [20]. In mice, PPP1CC knockout disrupts meiosis, leading to impaired spermatogenesis and infertility [17]. PPP1CC can also directly bind to the Ku70/Ku80 heterodimer and promote the formation of the DNA-PK holoenzyme, activating DNA-PKcs, enhancing non-homologous end joining (NHEJ)-mediated DNA repair, and contributing to radioresistance in nasopharyngeal carcinoma [18]. In the insulin-resistant HepG2 cell model, miR-140-5p has been shown to regulate glucose uptake and consumption by suppressing PPP1CC expression, alleviating insulin resistance [21]. Furthermore, *PPP1CC* has been identified as a candidate gene influencing chicken meat color traits, including lightness (L*) and redness (a*) [19]. Knockout of PPP1CC increases meat lightness and decreases myoglobin content, likely by regulating the expression of fast- and slow-twitch muscle fiber marker genes [19]. PPP1CC also regulates glycogen synthase dephosphorylation and inactivation and has been associated with skeletal muscle strength phenotypes [22]. However, the expression patterns, biological functions, and regulatory mechanisms of PPP1CC in adipocytes and adipose tissue remain largely unknown.

Our previous proteomic analysis of abdominal adipose tissue from Arbor Acres (AA) broilers identified PPP1CC as a potential interacting protein of NRF1 (data not published yet), although the underlying regulatory mechanism in adipogenesis remains unclear. NRF1 has been shown to directly bind to the P1 promoter of PPARγ and negatively regulate its transcriptional activity, inhibiting the differentiation of chicken preadipocytes [12]. As a multifunctional phosphatase with an undefined role in chicken adipogenesis, PPP1CC may enhance NRF1-mediated inhibition of PPARγ by modulating NRF1 expression or phosphorylation status. However, this regulatory mechanism and its functional relevance have not yet been experimentally validated.

Therefore, this study aimed to investigate the interaction between PPP1CC and NRF1 and to determine how PPP1CC regulates NRF1 phosphorylation and expression during chicken adipogenesis. It was further explored whether the PPP1CC–NRF1 axis enhances the inhibitory effect of NRF1 on the transcriptional activity of the *PPARγ* P1 promoter. Our results suggest that the PPP1CC–NRF1 signaling axis enhances NRF1-mediated negative regulation of PPARγ, inhibiting the differentiation of chicken preadipocytes.

## 2. Materials and Methods

### 2.1. Cells

The ICP1 and DF1 cell lines were kindly provided by the Poultry Research Group of Northeast Agricultural University.

### 2.2. Cell Culture and Differentiation of ICP1 Preadipocytes

DF1 cells were cultured in Dulbecco’s Modified Eagle Medium (DMEM) supplemented with 10% fetal bovine serum (FBS). ICP1 cells were maintained in DMEM/F12 complete medium containing 10% FBS [6].

For adipogenic differentiation, ICP1 cells were seeded uniformly into 6-well plates and cultured in high-glucose DMEM complete medium supplemented with 160 μM oleic acid. The culture medium was refreshed daily during the differentiation process.

### 2.3. Vectors

The plasmids pCMV-Myc, pCMV-HA, pCMV-HA-NRF1, and pGL3-Basic-PPARγ P1 were kindly provided by Northeast Agricultural University. The pCMV-Myc-PPP1CC plasmid was purchased from Miaolingbio Inc. (Wuhan, China).

### 2.4. Co-Immunoprecipitation (Co-IP)

Co-IP assays were performed according to the manufacturer’s instructions using the BeaverBeads™ Protein A/G Immunoprecipitation Kit (BEAVER, Suzhou, China). Adherent cells were lysed using IP Binding Buffer supplemented with protease inhibitors, followed by centrifugation to collect the supernatant containing the target antigens. Protein A/G magnetic beads were resuspended and washed twice with IP Binding Buffer using magnetic separation before use. Primary antibodies (anti-Myc) or control IgG were incubated with the pre-treated beads at room temperature to form bead–antibody complexes. After washing, the bead–antibody complexes were incubated with antigen-containing supernatants under appropriate conditions depending on antibody affinity. The complexes were then washed, transferred to new tubes, and eluted using either denaturing or non-denaturing elution methods. The eluted proteins were then analyzed by Western blot.

### 2.5. Western Blot Analysis

At predetermined time points, cultured cells were washed with ice-cold PBS and lysed in lysis buffer containing protease inhibitors. The lysates were centrifuged at 12,000 rpm for 5 min, and the supernatants were collected. The protein samples were mixed with 5× SDS loading buffer and denatured by boiling at 100 °C for 10 min. Protein samples and molecular weight markers (5 μL) were separated by SDS-PAGE at 100 V for approximately 1 h. The proteins were then transferred onto nitrocellulose (NC) membranes using wet transfer at 200 mA for 1 h. The membranes were blocked with 5% non-fat milk for 1 h and subsequently incubated overnight at 4 °C with the following primary antibodies: β-actin (1:5000; Beyotime, Beijing, China) and HA, Myc, NRF1, and phospho-threonine rabbit monoclonal antibodies (1:1000 each; Proteintech, Wuhan, China). After washing, the membranes were incubated for 1 h at room temperature with secondary antibodies: YSFluor™680 Goat Anti-Rabbit IgG (H + L) and YSFluor™680 Goat Anti-Mouse IgG (H + L) (1:10,000; Yeasen, Shanghai, China). Protein bands were visualized using the LI-COR Odyssey imaging system. Protein band intensities were quantified by ImageJ 1.54g software. To ensure accurate normalization, the intensity of each target protein band was divided by the intensity of the corresponding β-actin band from the same sample. All Western blot experiments were performed with three independent biological replicates (*n* = 3). Data are presented as the standard error of the mean (SEM) of these replicates.

### 2.6. Phosphorylated Protein Electrophoresis Analysis

For phosphorylated protein analysis, loading buffer (abs9941) was added to the protein samples at a 2:1 ratio, and the mixture was mixed thoroughly. The samples were denatured at 95 °C for 5–10 min. Approximately 20 μg of protein lysate was loaded onto Phos-tag SDS-PAGE precast gels and separated using 1× Tris-glycine-SDS electrophoresis buffer (abs9359). Electrophoresis was performed under constant current conditions (25–30 mA per gel or 50–60 mA for two gels simultaneously) until the bromophenol blue dye front reached the bottom of the separating gel. Before transfer, the gels were gently shaken in transfer buffer (abs90040) containing 10 mM EDTA for 10 min (repeated 1–2 times), followed by a 10 min wash with EDTA-free transfer buffer to improve transfer efficiency. Proteins were then transferred to membranes using wet transfer at 200 mA for 2 h. Subsequent detection steps were performed according to standard Western blot procedures.

### 2.7. Bioinformatics Prediction of Protein Dephosphorylation Sites

Potential dephosphorylation sites of the NRF1 protein targeted by the phosphatase PPP1CC were predicted using the online GPSD software (https://gpsd.biocuckoo.cn, accessed on 1 March 2025).

### 2.8. Construction of Dephosphorylation-Mimicking NRF1 Mutants

Based on bioinformatic predictions of potential phosphorylation sites, phospho-deficient (dephosphorylation-mimicking) NRF1 mutants were generated via gene synthesis. Specifically, ten predicted phosphorylation residues—Ser^44^, Ser^46^, Ser^52^, Ser^56^, Ser^102^, Ser^183^, Ser^257^, Thr^109^, Thr^118^, and Thr^249^—were substituted with alanine (Ala) to abolish their capacity for phosphorylation. The synthetic genes encoding these multi-site mutants were constructed, sequence-verified, and provided by Miaoling Biotechnology (Wuhan, China).

### 2.9. Quantitative Real-Time PCR (qRT-PCR) Analysis

Total RNA was reverse-transcribed into cDNA using the PrimeScript FAST RT Reagent Kit with gDNA Eraser (TransGen Biotech, Beijing, China). The *NONO* gene (non-POU domain-containing octamer-binding protein) was used as the internal reference gene for normalization of target gene expression. qRT-PCR was performed using TB Green^®^ Premix Ex Taq™ II (TransGen Biotech, Beijing, China). All reactions were conducted in triplicate to ensure reproducibility. Relative gene expression levels were calculated using the 2^−ΔΔCT^ method. The specific primer sequences used for qRT-PCR amplification are listed in Table 1.

### 2.10. Dual-Luciferase Reporter Assay

Luciferase activity was measured using the Dual-Lumi™ Dual-Luciferase Reporter Gene Assay Kit (Beyotime, Beijing, China) according to the manufacturer’s instructions. Briefly, 200 μL of lysis buffer was added to the cells, followed by incubation on a micro-oscillator at 450 rpm for 15 min to ensure complete cell lysis. After centrifugation, the supernatants were collected. For firefly luciferase activity detection, 100 μL of Dual-Lumi™ Firefly Luciferase Assay Reagent was added to a new 1.5 mL tube, followed by 20 μL of the cell lysate supernatant. After gentle pipetting (approximately 10 times), firefly luciferase (Fluc) activity was measured immediately. Subsequently, 100 μL of Dual-Lumi™ Renilla Luciferase Assay Reagent was added to the same tube, and Renilla luciferase (Rluc) activity was measured after gentle mixing. Relative luciferase activity was calculated as the ratio of Fluc to Rluc. Each experiment was independently repeated three times.

### 2.11. Chromatin Immunoprecipitation (ChIP) Analysis

ChIP assays were performed using the BeyoChIP™ Enzymatic Chromatin Immunoprecipitation Assay Kit with Protein A/G Magnetic Beads (Beyotime, Beijing, China). Approximately 1 × 10^7^ cells were fixed with 1% formaldehyde for 10 min at room temperature to cross-link protein–DNA complexes. The cross-linking reaction was quenched with pre-warmed glycine for 5 min. Cells were then washed with ice-cold 1× PBS, scraped into PBS containing protease inhibitor cocktail (PIC), and centrifuged at 2000× *g* for 5 min at 4 °C to collect cell pellets. Cell pellets were lysed using 1× Buffer A on ice for 10 min, followed by treatment with 1× Buffer B. Nuclei were collected by centrifugation and resuspended in 100 μL of 1× Buffer B. Chromatin was digested with 0.6 μL nuclease at 37 °C for 20 min, and the reaction was terminated with 10 μL of 0.5 M EDTA. The samples were then resuspended in 100 μL of 1× ChIP Buffer and subjected to sonication (99 pulses, 15 s on/30 s off, 7 cycles). After centrifugation at 9400× *g* for 10 min at 4 °C, the supernatants containing fragmented chromatin were collected. A portion (50 μL) of chromatin was treated with RNase A (37 °C, 30 min) and Proteinase K (65 °C, 2 h), purified, and verified by 1% agarose gel electrophoresis to confirm DNA fragments of 100–900 bp. DNA concentration was then measured. Approximately 2% of the chromatin was reserved as the input control and stored at −20 °C. The remaining chromatin (500 μL) was incubated overnight at 4 °C with either 5 μL of IgG or the specific target antibody. Subsequently, 30 μL of Protein G agarose beads were added and incubated for 4 h at 4 °C. The bead–chromatin complexes were washed three times with low-salt buffer and once with high-salt buffer (1 mL each, 4 °C, 10 min per wash), followed by centrifugation at 3400× *g* for 1 min. Chromatin was eluted using 150 μL of 1× ChIP elution buffer at 65 °C for 60 min with shaking (1200 rpm). The eluates and input samples were treated with 6 μL of 5 M NaCl and 2 μL of Proteinase K at 65 °C for 2 h to reverse the cross-linking of the protein–DNA complexes. The purified DNA was then analyzed by qPCR using specific primers (Table 1), and relative enrichment was calculated using the ΔΔCt method.

### 2.12. Oil Red O Staining

Differentiated ICP1 cells were fixed with 4% paraformaldehyde. After sequential washing with PBS and distilled water, the cells were stained with 0.5% Oil Red O solution for 15 min. Excess stain was removed using isopropanol.

The absorbance of lipid-associated staining was measured at 510 nm using a spectrophotometer to quantify intracellular lipid accumulation.

### 2.13. Immunofluorescence Analysis

Cells were seeded on glass coverslips in 24-well plates and cultured until reaching approximately 80% confluence. After treatment, cells were fixed with 4% paraformaldehyde for 15 min at room temperature and permeabilized with 0.1% Triton X-100 in PBS for 10 min. To block non-specific binding, cells were incubated with 5% BSA in PBS for 1 h. Subsequently, cells were incubated overnight at 4 °C with the primary antibody against NRF1 (Proteintech, Wuhan, China), Myc (Proteintech, Wuhan, China), HA (Proteintech, Wuhan, China) (diluted 1:1000 in 1% BSA). After washing three times with PBS, cells were incubated with a fluorescently labeled secondary antibody (TRITC-conjugated goat anti-mouse IgG and Alexa Fluor 488-conjugated goat anti-rabbit IgG, diluted 1:500) for 1 h at room temperature in the dark. Nuclei were counterstained with DAPI for 5 min. Fluorescence images were captured using a microscope (Olympus IX73) under identical exposure settings for all groups. For quantitative analysis, the mean fluorescence intensity (MFI) of NRF1 was measured in multiple random fields of view (at least 5 fields per replicate) using ImageJ. The final quantification represents the average MFI calculated from three independent biological experiments (n = 3). Cells showing non-specific background staining were excluded from the analysis.

### 2.14. Statistical Analysis

All experimental data are presented as the mean ± standard error of the mean (SEM). Each experiment was independently repeated three times (n = 3). Statistical comparisons between two independent groups were performed using Student’s *t*-test. For experiments involving multiple factors (e.g., treatment groups and different time points such as 24 h and 48 h), two-way analysis of variance (ANOVA) was used to evaluate the main effects of treatment and time, as well as their interaction. Where appropriate, repeated-measures ANOVA was applied for data collected from the same experimental batches across multiple time points. Post hoc comparisons were performed using Tukey’s multiple comparison test. All statistical analyses were conducted using Graphpad Prism 10.1.2 (GraphPad Software, Inc., San Diego, CA, USA).

## 3. Results

### 3.1. Identification of the Interaction Between Chicken PPP1CC and NRF1

**Figure 1:**
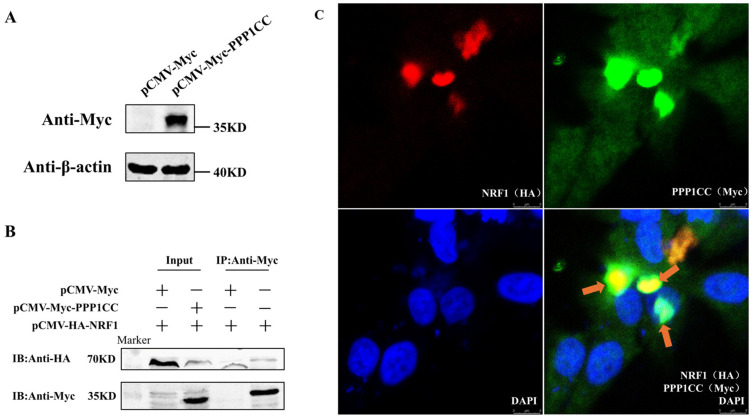
**Interaction between** **NRF1** **and** **PPP1CC**. (**A**) Validation of pCMV-Myc-PPP1CC overexpression efficiency by Western blot. DF1 cells were transfected with pCMV-Myc or pCMV-Myc-PPP1CC. Cells were collected 48 h after transfection, and PPP1CC overexpression was detected using an anti-Myc antibody. (**B**) Identification of the interaction between NRF1 and PPP1CC by co-immunoprecipitation (Co-IP). DF1 cells were co-transfected with pCMV-Myc or pCMV-Myc-PPP1CC together with pCMV-HA-NRF1. Immunoprecipitation was performed using an anti-Myc antibody, followed by Western blot analysis using anti-Myc and anti-HA antibodies. (**C**) Co-localization analysis by immunofluorescence. Chicken preadipocytes were co-transfected with pCMV-HA-NRF1 and pCMV-Myc-PPP1CC. Immunofluorescence staining was performed using mouse anti-HA and rabbit anti-Myc primary antibodies, followed by goat anti-mouse IgG (TRITC-conjugated) and goat anti-rabbit IgG (Alexa Fluor 488-conjugated) secondary antibodies. NRF1 is shown in red, PPP1CC in green, and nuclei were stained with DAPI (blue). Arrows indicate co-localization of NRF1 and PPP1CC (yellow, as indicated by the red color and arrows). Data were obtained from three independent experiments (*n* = 3).

To verify the potential interaction between PPP1CC and NRF1 [12], the eukaryotic expression plasmid pCMV-Myc-PPP1CC was first constructed, and its overexpression efficiency was confirmed by Western blot analysis (Figure 1A). The recombinant plasmid pCMV-Myc-PPP1CC and the laboratory-preserved eukaryotic expression vector pCMV-HA-NRF1 were co-transfected into DF1 cells to examine the co-localization and interaction between chicken PPP1CC and NRF1 proteins. Co-IP assays demonstrated that NRF1 could bind to PPP1CC (Figure 1B). Furthermore, immunofluorescence analysis revealed clear co-localization of NRF1 and PPP1CC proteins in both the nucleus and cytoplasm (Figure 1C). These results indicate that chicken NRF1 interacts with PPP1CC in both the nucleus and cytoplasm.

### 3.2. Dephosphorylation of NRF1 by Chicken PPP1CC

**Figure 2:**
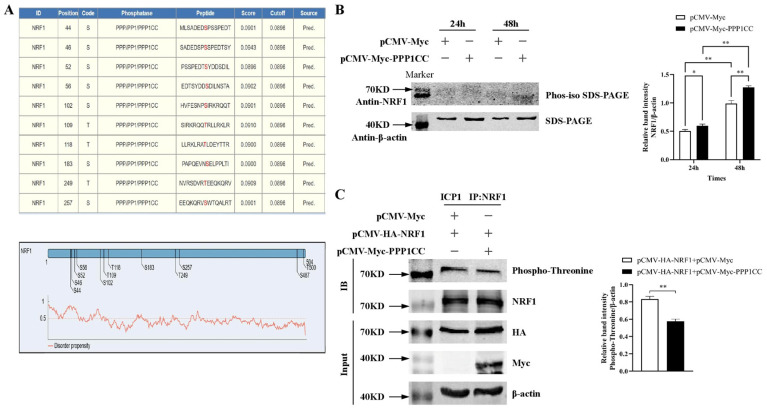
**PPP1CC** **overexpression promotes** **NRF1** **dephosphorylation.** (**A**) Prediction of PPP1CC-targeted dephosphorylation sites on NRF1 using GPSD analysis. Potential phosphorylation sites include Ser^44^/^46^/^52^/^56^/^102^/^183^/^257^ and Thr^109^/^118^/^249^. Structural schematic and disorder plots are shown. (**B**) Phos-iso SDS-PAGE analysis showing increased electrophoretic mobility of NRF1 after PPP1CC overexpression, indicating NRF1 dephosphorylation at 48 h following oleic acid induction. Data are presented as mean ± SEM (*n* = 3). Statistical analysis was performed using two-way ANOVA (* *p* < 0.05, ** *p* < 0.01). (**C**) Immunoprecipitation and Western blot analysis showing that PPP1CC overexpression reduces threonine phosphorylation of NRF1. Statistical analysis was performed using Student’s *t*-test (** *p* < 0.01).

Since PPP1CC is a protein phosphatase that dephosphorylates serine and threonine residues on target proteins [23], we next investigated whether PPP1CC could regulate the phosphorylation status of NRF1. Potential PPP1CC-mediated dephosphorylation sites on NRF1 were predicted using the online software GPSD (https://gpsd.biocuckoo.cn, accessed on 1 March 2025). The results indicated that Ser^44^/^46^/^52^/^56^/^102^/^183^/^257^ and Thr^109^/^118^/^249^ of NRF1 were potential dephosphorylation sites targeted by PPP1CC (Figure 2A), suggesting that PPP1CC may induce NRF1 dephosphorylation.

We then examined the effect of PPP1CC on NRF1 phosphorylation. Because phospho-specific antibodies against NRF1 were not available, Western blot analysis was performed using NRF1 antibodies in combination with Phos-tag SDS-PAGE (Glass gel, Absin) to evaluate the effect of PPP1CC overexpression on NRF1 phosphorylation. ICP1 cells were transfected with either an empty vector or a PPP1CC expression plasmid and subsequently induced with 160 μM oleic acid for 24 h or 48 h. Cell lysates were then analyzed by Phos-tag SDS-PAGE Western blot using NRF1 antibodies. The results showed that after 24 h of oleic acid induction, PPP1CC overexpression did not alter the electrophoretic mobility of NRF1 compared with the control group (empty vector). However, at 48 h post-induction, PPP1CC overexpression increased the electrophoretic mobility of NRF1, with the NRF1 band shifting downward relative to the control group, indicating that PPP1CC overexpression promoted NRF1 dephosphorylation (Figure 2B). The NRF1 protein bands in both the control group and the PPP1CC-overexpression group at 48 h after oleic acid induction exhibited a clear downward shift compared with those observed at 24 h. This finding suggests that NRF1 undergoes progressive dephosphorylation during preadipocyte differentiation (Figure 2B).

To further determine whether PPP1CC affects threonine phosphorylation of NRF1, a universal phospho-threonine rabbit monoclonal antibody (AG0419) was used. ICP1 cells were co-transfected with either an empty vector or pCMV-Myc-PPP1CC together with pCMV-HA-NRF1, followed by immunoprecipitation using NRF1 antibodies. The results confirmed successful enrichment of the NRF1 protein. Compared with the control group, PPP1CC overexpression significantly reduced threonine phosphorylation of NRF1 (Figure 2C), further supporting that PPP1CC promotes NRF1 dephosphorylation.

### 3.3. Effect of PPP1CC on NRF1 Expression

**Figure 3:**
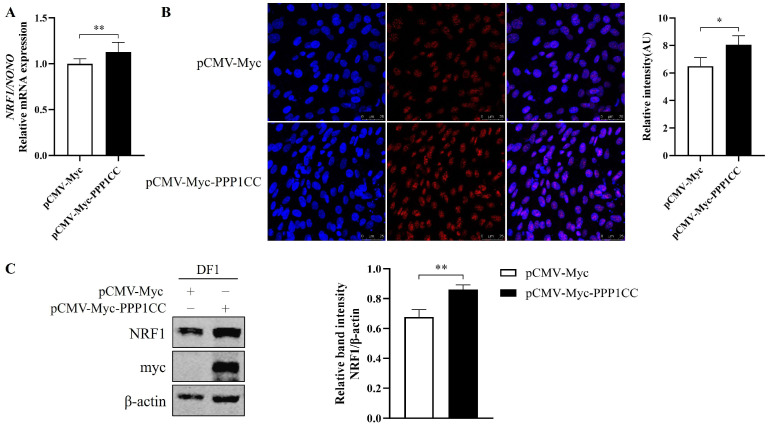
**Effect of** **PPP1CC** **overexpression on endogenous** **NRF1** **expression.** (**A**) Chicken preadipocytes were transfected with pCMV-Myc-PPP1CC or pCMV-Myc, and NRF1 mRNA levels were measured 48 h after transfection. (**B**) Immunofluorescence analysis of NRF1 expression in chicken preadipocytes transfected with pCMV-Myc-PPP1CC or pCMV-Myc for 48 h. Cells were stained using an anti-NRF1 antibody. (**C**) Western blot analysis of NRF1 protein expression in DF1 cells transfected with pCMV-Myc-PPP1CC or pCMV-Myc. The Myc tag and β-actin were used as controls for transfection efficiency and protein loading, respectively. Relative NRF1 protein levels were quantified as the NRF1/β-actin ratio (right panel). Data are representative of three independent experiments (*n* = 3) and are presented as mean ± SEM. Statistical analysis was performed using Student’s *t*-test (* *p* < 0.05, ** *p* < 0.01 vs. control).

To determine whether PPP1CC regulates NRF1 expression, the effect of PPP1CC overexpression on endogenous NRF1 levels was investigated in chicken preadipocytes. Cells were transfected with either an empty vector or the pCMV-Myc-PPP1CC expression plasmid. The results showed that PPP1CC overexpression significantly upregulated both the mRNA and protein expression levels of NRF1 compared with the control group. Specifically, qPCR analysis revealed a significant elevation in *NRF1* mRNA levels (Figure 3A). Consistently, immunofluorescence staining (Figure 3B) and Western blot analysis (Figure 3C) demonstrated a significant increase in NRF1 protein expression in PPP1CC-overexpressing cells. These results indicate that PPP1CC positively regulates NRF1 expression at both the transcriptional and protein levels.

### 3.4. PPP1CC Enhances NRF1-Mediated Suppression of the PPARγ P1 Promoter via Dephosphorylation

**Figure 4:**
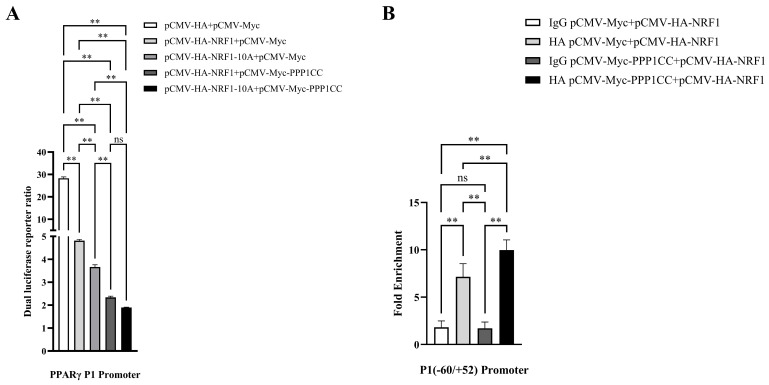
**Effect of** **PPP1CC** **overexpression on** **NRF1-mediated transcriptional activity of the** **PPARγ** **promoter.** (**A**) Dual-luciferase reporter assay showing the effect of PPP1CC overexpression on NRF1-mediated transcriptional activity of the *PPARγ* P1 promoter. (**B**) Chromatin immunoprecipitation followed by quantitative PCR (ChIP–qPCR) analysis assessing the effect of PPP1CC overexpression on the binding of NRF1 to the *PPARγ* P1 promoter. Data were obtained from three independent experiments (*n* = 3) and are presented as mean ± SEM. Statistical analysis was performed using one-way analysis of variance (ANOVA). Significance levels are denoted as ** *p* < 0.01 vs. control, ns, not significant.

To further determine whether PPP1CC regulates the transcriptional activity of NRF1 through specific phosphorylation sites, this study focused on several key amino acid residues of NRF1 (Ser^44^/^46^/^52^/^56^/^102^/^183^/^257^ and Thr^109^/^118^/^249^). It was hypothesized that PPP1CC-mediated dephosphorylation of these residues might affect NRF1-mediated transcriptional repression of the *PPARγ* P1 promoter. To test this hypothesis, point mutations were introduced into NRF1 to generate dephosphorylation-mimicking mutants in which the above serine and threonine residues were substituted with alanine (NRF1-10A). These mutants were analyzed using a dual-luciferase reporter assay system to evaluate their regulatory effects on *PPARγ* P1 promoter activity. Luciferase reporter assays showed that the dephosphorylation-mimicking NRF1 mutant more strongly repressed the *PPARγ* P1 promoter. PPP1CC overexpression significantly enhanced this inhibition by NRF1, with no significant difference between wild-type and phosphorylation-deficient (dephosphorylation-mimicking) NRF1. These results suggest that PPP1CC may regulate NRF1 function through the modulation of the phosphorylation status of these Ser/Thr clusters. However, whether these specific residues serve as direct substrates for PPP1CC remains to be determined. Moreover, ChIP-qPCR assays revealed that PPP1CC overexpression resulted in a significant increase in NRF1 occupancy at the *PPARγ* P1 promoter region compared to the control group (Figure 4B).

### 3.5. Effects of PPP1CC on Differentiation of Chicken Preadipocytes

**Figure 5:**
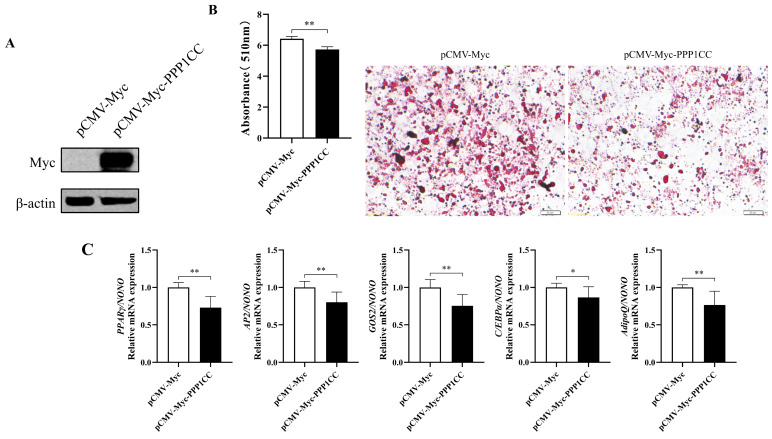
**PPP1CC** **overexpression inhibits differentiation of chicken preadipocytes.** (**A**) Western blot analysis verifying overexpression of pCMV-Myc-PPP1CC. (**B**) Effects of PPP1CC overexpression on lipid droplet accumulation during ICP1 differentiation. Oil Red O staining images and quantitative analysis of lipid accumulation were obtained from pCMV-Myc and pCMV-Myc-PPP1CC-transfected ICP1 cells at 48 h. (**C**) qRT-PCR analysis of adipogenic marker genes (*PPARγ*, *AP2*, *GOS2*, *CEBPα*, and *AdipoQ*) in ICP1 cells transfected with pCMV-Myc (white bars) or pCMV-Myc-PPP1CC (black bars) at the indicated differentiation time points. All experiments were performed independently three times (*n* = 3). Data are presented as mean ± SEM. Statistical significance was defined as * *p* < 0.05, ** *p* < 0.01.

Overexpression of PPP1CC significantly enhanced the inhibitory effect of NRF1 on the *PPARγ* P1 promoter. Based on these results, it was hypothesized that PPP1CC interacts with NRF1 to promote NRF1 dephosphorylation and increase *NRF1* mRNA and protein levels, enhancing the negative regulation of the *PPARγ* P1 promoter by NRF1 and ultimately affecting the differentiation of chicken preadipocytes. To test this hypothesis, PPP1CC was overexpressed in chicken preadipocytes, followed by induction of differentiation with oleic acid for 48 h. Western blot analysis confirmed successful overexpression of PPP1CC, as PPP1CC protein expression was clearly detected in the PPP1CC-overexpressing group but not in the control group (Figure 5A). After 48 h of differentiation induction, PPP1CC overexpression significantly reduced lipid droplet accumulation during chicken preadipocyte differentiation (*p* < 0.01; Figure 5B). The analysis of adipogenic marker genes further revealed that PPP1CC overexpression significantly inhibited the expression of key differentiation markers, including *PPARγ*, *AP2*, *GOS2*, *CEBP/α*, and *AdipoQ* (*p* < 0.05 or *p* < 0.01; Figure 5C). These results indicate that PPP1CC significantly inhibits differentiation of ICP1 chicken preadipocytes.

## 4. Discussion

Excessive abdominal fat deposition represents a major limitation on production efficiency and product quality in broiler chicken farming. Understanding the molecular regulatory network underlying adipogenesis is therefore essential for improving broiler breeding strategies [24,25]. In this study, it was demonstrated, for the first time, that the protein phosphatase PPP1CC interacts with the transcription factor NRF1 and regulates NRF1 expression via a dephosphorylation-dependent mechanism. This process enhances the inhibitory effect of NRF1 on PPARγ, a key regulator of adipogenesis, suppressing the differentiation of chicken preadipocytes. These results reveal a novel, previously unrecognized phosphatase–transcription factor regulatory pathway involved in chicken adipogenesis and provide new insight into the species-specific molecular mechanisms underlying avian fat deposition.

PPP1CC, an important member of the PP1 phosphatase family, is known to participate in various physiological processes, including spermatogenesis, DNA repair, and muscle fiber-type regulation; however, its role in adipogenesis has not been reported previously [16,19]. This study found that PPP1CC overexpression significantly inhibited the differentiation of chicken preadipocytes. PPP1CC promoted dephosphorylation of NRF1, significantly increasing NRF1 expression. This regulatory pattern is particularly important because it demonstrates that PPP1CC can regulate both the phosphorylation status and the expression level of a key transcription factor involved in adipogenesis. These findings suggest that PPP1CC functions as a signaling node capable of integrating dephosphorylation events to precisely regulate transcriptional activity in adipocytes. Previous studies have shown that NRF1 phosphorylation status plays an important regulatory role in controlling its activity [15]. For example, CDK2 interacts with NRF1 to phosphorylate it at two serine residues, inhibiting NRF1’s DNA-binding activity [15]. In mouse germ cells, deletion of *Cdk2* increases the expression of *Ehmt1*, a downstream target gene of NRF1, indicating that the CDK2–NRF1–Ehmt1 axis participates in regulating H3K9 methylation dynamics during meiotic prophase I [15]. Moreover, TBK1-mediated phosphorylation of NRF1 at Ser318 inactivates the NRF1–TFAM axis, affecting mitochondrial biogenesis and suppressing innate antiviral immunity through mtDNA release [13]. These findings highlight the key role of phosphorylation in modulating NRF1 function across multiple biological contexts.

Combined bioinformatic predictions and phosphorylation-specific electrophoresis analyses indicate that PPP1CC targets multiple serine and threonine residues within NRF1 for dephosphorylation. Notably, this dephosphorylation event coincides with the timeline of oleic acid–induced preadipocyte differentiation, suggesting a critical temporal link between NRF1 modification and adipogenic regulation. Based on these observations, we propose a model in which PPP1CC likely regulates NRF1 function by modulating the phosphorylation status of these specific Ser/Thr clusters, rather than acting on isolated, definitively mapped sites.

NRF1 has previously been identified as a negative regulator of chicken adipogenesis, functioning by directly binding to the P1 promoter of the *PPARγ* gene and suppressing its transcriptional activity [12]. Our results further demonstrate that PPP1CC overexpression increases NRF1 occupancy at the *PPARγ* P1 promoter, which may likely be attributed to the PPP1CC-mediated upregulation of total NRF1 protein levels. Our results further demonstrate that PPP1CC significantly enhances the inhibitory effect of NRF1 on the *PPARγ* gene. Thus, the PPP1CC–NRF1–PPARγ regulatory axis identified in this study represents a new upstream regulatory axis of the adipogenic transcriptional network. Given that PPARγ and C/EBPα are widely recognized as key drivers of adipogenesis that coordinate the expression of lipid metabolism-related genes [26], modulation of PPARγ activity by upstream regulators such as PPP1CC may represent an important mechanism controlling adipocyte differentiation.

The existence of this regulatory axis may provide a new molecular mechanism that may help explain individual variation in fat deposition among broilers. Differences in PPP1CC expression levels among individuals may affect NRF1 dephosphorylation and expression, altering PPARγ repression and ultimately affecting adipogenic potential. These observations suggest that *PPP1CC* could be a candidate gene for molecular breeding strategies to improve fat deposition traits in broiler chickens by modulating NRF1 expression. The PPP1CC–NRF1 axis identified herein is a novel target for developing targeted regulatory strategies. As PPP1CC also has homologous genes in mammals, the regulatory mechanism identified here may also provide insights for comparative studies of adipose metabolism across species.

Despite these findings, several limitations should be acknowledged. It is important to note that our study utilized a multi-site mutant (NRF1–10A) to assess the collective role of these phosphorylation sites. While the lack of additive effect upon PPP1CC overexpression strongly implies a shared regulatory pathway, we cannot rule out the possibility that only a subset of these ten residues are direct targets of PPP1CC, or that PPP1CC acts indirectly via upstream kinases. Future work employing single-site mutagenesis and in vitro phosphatase assays will be required to definitively map the direct dephosphorylation sites.

Our study demonstrates that the PPP1CC–NRF1 axis exerts its specific regulatory function at 48 h of differentiation, characterized by prominent NRF1 dephosphorylation and adipogenic inhibition. We acknowledge a limitation, the lack of continuous monitoring beyond this stage. Given the multi-stage nature of adipogenesis, it is imperative to conduct dynamic functional studies in the future. Mapping the temporal trajectory of PPP1CC activity (e.g., at 72 h, 96 h, and maturation) is essential to distinguish whether this dephosphorylation serves as a transient “switch” for commitment or a sustained mechanism for terminal maturation. Such dynamic functional analysis is crucial to fully construct the temporal landscape of the PPP1CC–NRF1 network.

While this study elucidates the molecular mechanism of the PPP1CC–NRF1 axis in chicken preadipocytes, we acknowledge that direct validation in in vivo chicken adipose tissues was not performed. Consequently, while our in vitro data provide a strong mechanistic foundation, the direct association between this axis and obesity-related phenotypes in living chickens remains to be fully established. Future in vivo investigations are warranted to confirm whether the regulatory patterns observed in our cellular models accurately reflect the physiological status of adipose tissue during avian obesity development.

In summary, this study demonstrates that PPP1CC increases NRF1 expression through a dephosphorylation-dependent mechanism, enhancing NRF1-mediated repression of PPARγ and ultimately suppressing chicken preadipocyte differentiation. Based on these results, it was hypothesized that PPP1CC binds to the transcription factor NRF1 and regulates its expression by dephosphorylation, modulating the transcriptional activity of NRF1 target genes and inhibiting PPARγ-driven adipogenic signaling pathways. Elucidation of this novel PPP1CC–NRF1 regulatory mechanism expands the current understanding of adipogenic regulation in chickens and fills an important gap regarding the role of PPP1CC in fat metabolism. This study provides valuable insights to improve broiler production efficiency.

## 5. Conclusions

The phosphatase PPP1CC interacts with the transcription factor NRF1, dephosphorylating it and enhancing NRF1 expression. This process promotes the transcriptional repression of the target gene PPARγ by NRF1, inhibits PPARγ-mediated adipogenesis-related signaling pathways, and suppresses the differentiation of chicken preadipocytes. The study elucidates the regulatory mechanism of the PPP1CC–NRF1–PPARγ axis in chicken preadipocytes and identifies novel candidate targets. It provides a theoretical foundation for molecular breeding strategies for fat-deposition traits in broilers.

## Figures and Tables

**Table 1:** List of primers used in this study.

Primer	Sequence (5′ to 3′)
PPARγ-F	GTGCAATCAAAATGGAGCC
PPARγ-R	CTTACAACCTTCACATGCAT
AP2-F	ATGTGCGACCAGTTTGT
AP2-R	TCACCATTGATGCTGATAG
GOS2-F	CGGGGCGAAAGAGCTGAG
GOS2-R	AGCACGTACAGCTTCACCAT
C/EBPα-F	GGAGCAAGCCAACTTCTACGC
C/EBPα-R	CTCGTTCTCGCAGATGTCGC
AdiPoQ-F	GCAACAACAACGGGGTCT
AdiPoQ-R	AGGGGAATTTTCTGGTACATAG
NONO-F	AGAAGCAGCAGCAAGAAC
NONO-R	TCCTCCATCCTCCTCAGT
NRF1-F	ACCCATCCATCCGTAAGAG
NRF1-R	CTTGCGTACCACATTCTCC

## Data Availability

The original contributions presented in this study are included in the article. Further inquiries can be directed to the corresponding author.

## Abbreviations

The following abbreviations are used in this manuscript:

NRF1	Nuclear Respiratory Factor 1
PPARγ	peroxisome proliferator-activated receptor gamma
PPP1CC	Protein Phosphatase 1 Catalytic Subunit Gamma
Co-IP	Co-immunoprecipitation
FBS	fetal bovine serum
Fluc	firefly luciferase
Rluc	Renilla luciferase
SEM	standard error of the mean
ANOVA	one-way analysis of variance
AA	Arbor Acres
IP	Immunoprecipitation
IF	Immunofluorescence
